# IOL Power Calculation after Corneal Refractive Surgery

**DOI:** 10.1155/2014/658350

**Published:** 2014-07-21

**Authors:** Maddalena De Bernardo, Luigi Capasso, Luisa Caliendo, Francesco Paolercio, Nicola Rosa

**Affiliations:** ^1^Department of Medicine and Surgery, University of Salerno, Via Salvatore Allende1, Baronissi, 84081 Salerno, Italy; ^2^U.O.C. Corneal Transplant Unit, Pellegrini Hospital, 80100 Naples, Italy; ^3^U.O.C. Eye Day Surgery, De Luca e Rossano Hospital, 80069 Vico Equense, Italy

## Abstract

*Purpose*. To describe the different formulas that try to overcome the problem of calculating the intraocular lens (IOL) power in patients that underwent corneal refractive surgery (CRS). *Methods*. A Pubmed literature search review of all published articles, on keyword associated with IOL power calculation and corneal refractive surgery, as well as the reference lists of retrieved articles, was performed. *Results*. A total of 33 peer reviewed articles dealing with methods that try to overcome the problem of calculating the IOL power in patients that underwent CRS were found. According to the information needed to try to overcome this problem, the methods were divided in two main categories: 18 methods were based on the knowledge of the patient clinical history and 15 methods that do not require such knowledge. The first group was further divided into five subgroups based on the parameters needed to make such calculation. *Conclusion*. In the light of our findings, to avoid postoperative nasty surprises, we suggest using only those methods that have shown good results in a large number of patients, possibly by averaging the results obtained with these methods.

## 1. Introduction

Since the introduction of excimer laser treatment in the field of refractive surgery, several problems such as incorrect measurement of the intraocular pressure and intraocular lens (IOL) power calculation, have been pointed out [1–12].

It is well known that the calculation of the power of the IOL to be implanted in patients undergoing cataract surgery is mainly based on the measurement of corneal power, on axial length, and on the forecast of the actual position of the lens after surgery [13–21].

In eyes that underwent corneal refractive surgery (CRS), all the routinely used methods to measure corneal power do not guarantee the same accuracy compared to the same measurements in naive eyes. It has been extensively demonstrated that after myopic refractive surgery (PRK, LASIK, RK) both keratometry and corneal topography tend to overestimate the corneal power [22–35].

For this reason, if a patient develops a cataract after these procedures, using the current values of keratometry readings (*K*), the IOL power could be underestimated and the patient may have a considerable risk of becoming hyperopic [36–42].

Hyperopia after cataract surgery is not only a surprising result, but also a real disaster in terms of refractive outcome as pseudophakic eyes have lost their accommodative ability. In severe cases it may be necessary to remove and to replace the implanted IOL.

The purpose of the present paper is to describe the different available techniques to improve the accuracy of the IOL power calculation after refractive procedures.

## 2. Methods

An accurate Pubmed database search review of the recent literature from 2000 to nowadays was performed to identify all potentially relevant published studies. The search strategy used the following keywords: IOL power calculation and refractive surgery. The literature search was conducted between January and the march 2014. The references cited in retrieved articles were also scanned for any additional relevant studies.

From this research 33 papers have been found.

All the authors of these papers agree that the main problem in the IOL power calculation after CRS is due to the incorrect keratometry readings.

After an accurate reading of these papers the collected methods have been divided in two main categories: methods based on the knowledge of the patient clinical history and methods that do not require the patient's medical history.

Moreover the methods based on the knowledge of the patient clinical history have been further divided into four subgroups based on the parameters needed to make such a calculation:

1. the preoperative refraction and keratometry plus the postrefractive surgery refraction,
2. the pre- and postrefractive surgery refractions,
3. the preoperative keratometry,
4. the preoperative keratometry and refraction,
5. the prerefractive surgery refraction.

## 3. Results

According to this classification, the papers assigned to the different categories were as follows.

### 3.1. Methods Based on the Knowledge of the Patient Clinical History

#### 3.1.1. Knowledge of the Preoperative Keratometry and Refraction and Postrefractive Surgery Refraction

The so-called “Medical history method”, described in 1989 by Eiferman [43] and Holladay [44], which later was modified by Hoffer [45], belongs to this group. With this method the *K* value is calculated subtracting the change in refraction, induced by the treatment, to the mean preoperative corneal power:

(1) $$\begin{array}{c} \;K_{\text{eff}}=K_{\text{pre}}-\Delta \text{Ref}, \end{array}$$

where *K*_eff_ is corneal power to be included in calculation formulas, *K*_pre_ is preoperative mean corneal power, and ΔRef is change of refraction measured as spherical equivalent.

For many years this method has been considered to be the gold standard, but today it is considered outdated.

#### 3.1.2. Knowledge of the Preoperative and Postrefractive Surgery Refraction

The following methods belong to this group.

Camellin and Calossi [46] proposed two formulas, according to the different treatment (PRK or RK) in which the IOL power is calculated utilizing a corneal radius, function of the preoperative one, modified taking into account the surgically induced refractive change. The formulas are the following:

(2) $$\begin{array}{c} P=\frac{1336\left(4R_{\text{adj}}-L\right)}{\left(L-{\text{ACD}}_{\text{post}}\right)\left(4R_{\text{adj}}-{\text{ACD}}_{\text{post}}\right)}, \end{array}$$

where *P* is power of the IOL to be implanted, *L* is axial length, ACD_post_ represents the actual position of the IOL, and *R*_adj_ is radius of curvature after refractive surgery, modified depending on whether the individual has undergone incisional surgery:

(3) $$\begin{array}{c} R_{\text{adj}}=\frac{0.3319\times R}{n_{\text{adj}}-1}=\frac{0.3319\times R}{0.00096\times \text{SIRC}+0.3319} \end{array}$$

or photorefractive surgery:

(4) $$\begin{array}{c} R_{\text{adj}}=\frac{0.3316\times R}{n_{\mathrm{rel}}-1}=\frac{0.3319\times R}{0.0013\times \text{SIRC}+0.3319}, \end{array}$$

where SIRC is refractive changes induced by surgery.

The authors tested the formula in 15 eyes undergoing IOL implantation after cataract surgery, with a mean postoperative error of +0.28*D* ± 0.66*D* ranging from −1.04*D* to +1.58*D* with 9 eyes in the range of ±0.5*D*, 12 eyes in the range of ±1*D*, and 15 eyes in the range of ±2*D*.

Chen and Hu [47] proposed two formulas, for two different devices utilized for measuring the corneal power, namely, (1) the Topcon CR 3000 autokeratometer and (2) the TMS1 Corneal topographer.

(1) consider
  (5) $$\begin{array}{c} \Delta \text{Auto}\;K=0.7397\times \Delta \text{ES}+0.3778, \end{array}$$

where ΔAuto *K* is change in corneal power after corneal refractive surgery, ΔES is pre- and postoperative refractive change.

(2) consider
  (6) $$\begin{array}{c} \Delta K\;\text{Central}=0.9183\times \Delta \text{ES}-0.0204, \end{array}$$

where Δ*K* Central is change in Central keratometry, ΔES is change in pre- and postoperative refraction.

In both cases the change in corneal power after refractive surgery is based on a linear correlation with the change in refraction induced by refractive surgery. The difference between these two values is then subtracted from the postoperative *K* to obtain the actual *K*.

Diehl et al. [48, 49] suggest a formula where the change in refraction is utilized to calculate the target refractive error to achieve emmetropia:

(7) Target  postoperative  refractive  error  (D)  to  achieve  emmetropia  during  IOL  power  calculation =−0.018∗(MRSE  Change)∗(MRSE  Change)  +0.192∗(MRSE  Change)−0.062,

where MRSE change is manifest refraction spherical equivalent change in diopters.

The outcomes in 97% of the 32 examined eyes fell within ±1.00 *D* of the value predicted by this formula.

Feiz et al. [50, 51] suggested two different regression formulas to be used depending on whether the patient underwent a myopic or hyperopic LASIK treatment, in which the difference in refraction induced by the treatment is subtracted from the IOL power traditionally calculated:

Myopic LASIK:

(8) $$\begin{array}{c} \text{IOL}\;\;\text{imp}=\text{IOL}\;\;\text{calc}-0.231+\left(0.595\times \Delta \text{ES}\right), \end{array}$$

Hyperopic LASIK:

(9) $$\begin{array}{c} \text{IOL}\;\;\text{imp}=\text{IOL}\;\;\text{calc}+0.751-\left(0.862\times \Delta \text{ES}\right), \end{array}$$

where IOL imp is power of IOL to be implanted, IOL calc is the power of IOL calculated by the traditional method, and ΔES is difference in refraction before and after refractive surgery.

The authors tested the formula in 19 eyes undergoing IOL implantation after cataract surgery, with a mean postoperative refractive error of −0.375 ± 2.3*D* ranging from −2*D* to +1.25*D* with 12 eyes (63.2%) in the range of ±0.5*D*, 16 eyes (84.2%) in the range of ±1*D*, and 19 eyes (100%) in the range of ±1.5*D*.

Hamed et al. [52] proposed two formulas, for two different devices utilized for measuring the corneal power, namely, the Bausch and Lomb keratometer and the EyeSys topographer: in both cases the corneal power is calculated with a regression formula, subtracting the RS-induced change in refraction from the mean postoperative corneal power:

(a) (10) $$\begin{array}{c} K_{\text{post-adj}}=K_{\text{post}}-0.24\times \left(\Delta \text{Rif}\right)+0.15, \end{array}$$
(b) (11) $$\begin{array}{c} \text{EffR}{\text{P}}_{\text{post-adj}}=\text{EffR}{\text{P}}_{\text{post}}-0.15\left(\Delta \text{Rif}\right)-0.05, \end{array}$$

where *K*_post-adj_ is corneal power to be included in calculation formulas, *K*_post_ is average postoperative corneal power obtained by keratometry, ΔRif is pre- and postoperative refractive change measured as spherical equivalent, EffRP_post-adj_ is corneal power to be included in calculation formulas, and EffRP_post_ is average postoperative corneal power using the parameter EffRP from EyeSys topographer.

The authors do not provide data to support the reliability of their method.

Jarade et al. [53] suggested a formula in which the *K* value is calculated from the ratio between the effective treatment and the anterior corneal radius of curvature:

(12) $$\begin{array}{c} K\text{-reading}=\frac{\left(rN-1\right)}{R_{a}}, \end{array}$$

where *R*_*a*_ is radius of anterior corneal curvature measured in meters, *rN* = 0.0014 ∗ Δ + 1.3375, and Δ is amount of myopic ablation.

The authors do not provide data to support the reliability of their method.

S. Masket and S. E. Masket [54] suggested a formula related to the effective treatment at the corneal apex, to calculate a factor to be added to the calculated IOL power:

(13) $$\begin{array}{c} \text{IOL}\;\text{power}\;\text{add}=\left(\text{LSE}*-0.326\right)+0.101, \end{array}$$

where IOL power add is power of IOL to be added and LSE is effective treatment at the corneal apex.

The author provides no data to support the reliability of the method.

Rosa et al. [42] proposed the following formula: *y* = 0.7615*x* − 0.6773, where *x* is difference in refraction at the corneal plane and *y* is keratometric difference evaluated with the IOL Master. To obtain the corrected *K* the difference between *x* and *y* has to be subtracted from the values of measured postoperative *K*. The authors do not provide data to support the reliability of their method.

Stakheev and Balashevich [55] described a formula in which there is a linear correlation between the corneal power correcting factor and the effective treatment, utilizing different constants, varying depending on the performed treatment and the device used to measure the corneal power:

(14) $$\begin{array}{c} Y=aX-b, \end{array}$$

where *Y* is corneal power correcting factor, *X* is effective treatment, and *a* and *b* vary depending on the type of refractive surgery performed and the equipment used to measure the corneal power.

In the case of LASIK one has the following:

- Humphrey autokeratometer:
  1. *a* = 0.225,
  2. *b* = 0.3893,
- Grand Seiko GR3100 autokeratometer:
  1. *a* = 0.3356,
  2. *b* = 0.453,
- Sim *K* with the Humphrey topographer:
  1. *a* = 0.2876,
  2. *b* = 0.5402,
- Average Corneal Power with the Humphrey topographer:
  1. *a* = 0.1468,
  2. *b* = 0.4468.

In the case of PRK one has the following:

- Humphrey autokeratometer:
  1. *a* = 0.2537,
  2. *b* = 0.5322,
- Grand Seiko GR3100 autokeratometer:
  1. *a* = 0.3701,
  2. *b* = 0.89,
- Sim *K* with the Humphrey topographer:
  1. *a* = 0.3341,
  2. *b* = 0.7857,
- Average Corneal Power Humphrey topographer:
  1. *a* = 0.2325,
  2. *b* = 0.643.

In the case of RK one has the following:

- Humphrey autokeratometer:
  1. *a* = 0.0256,
  2. *b* = 1.0957,
- Grand Seiko GR3100 autokeratometer:
  1. *a* = 0.2572,
  2. *b* = 1.3328,
- Sim *K* with the Humphrey topographer:
  1. *a* = 0.2189,
  2. *b* = 1.4481,
- Average Corneal Power Humphrey topographer:
  1. *a* = 0.0479,
  2. *b* = 0.7457.

#### 3.1.3. Knowledge of the Preoperative Keratometry

The following methods belong to this group.

Aramberri [56, 57], assuming that the error in the calculation is due to an incorrect estimation of the actual position of the IOL, suggested the so-called double-*K* method, which relies on the use of both preoperative and postoperative *K*, which are needed to calculate the effective lens position:

(15) IOLemme=[1000∗na∗(na∗rpost−0.333∗LOPT)][(LOPT−ACDest)∗(na∗rpost−0.333∗ACDest)],

where *n*_*a*_ = 1.336, *r*_post_ is radius of curvature after refractive surgery, LOPT = *L* + (0.65696 − 0.02029∗*L*), and *L* is axial length; ACD_est_ is estimated anterior chamber depth that requires knowledge of preoperative radius of curvature.

To prove the validity of the formula the author tested it in 9 eyes undergoing IOL implantation after cataract surgery, obtaining a mean postoperative refractive error of 0.43*D* ± 0.44*D* with a range from −0.56 to1.47*D* with 6 eyes (66.66%) in the range of ±0.5*D* and 8 eyes (88.88%) in the range of ±1*D*.

Jarade and Tabbara [58] suggested, to calculate the corneal power, the following formula:

(16) $$\begin{array}{c} K_{\text{postop}}=K_{\text{prepop}}-\left[\frac{\left(N_{c}-1\right)*\left(R_{a\text{-postop}}-R_{a\text{-preop}}\right)}{\left(R_{a\text{-postop}}*R_{a\text{-preop}}\right)}\right], \end{array}$$

where *K*_postop_ is corneal power to be included in the formula to calculate the IOL, *K*_prepop_ is corneal power before corneal refractive surgery, *N*_*c*_ is cornea's index of refraction (1.376), *R*_*a*-postop_ is radius of curvature of the anterior surface of the cornea after refractive surgery, and *R*_*a*-preop_ is radius of curvature of the anterior surface of the cornea before refractive surgery.

The authors do not provide data to support the reliability of their method.

Seitz et al. [59] suggested calculating the corneal power by subtracting from the preoperative corneal power a number derived from the inverse of the pre- and postoperative keratometric values:

(17) $$\begin{array}{c} K_{\text{calc-ex}}=K_{\text{pre}}-\left[\frac{0.376}{\left(0.3313*K_{\text{pre}}\right)}-\frac{0.376}{\left(0.3313*K_{\text{post}}\right)}\right], \end{array}$$

where *K*_calc-ex_ is keratometric value to be included in the formula; *K*_pre_ is keratometric value prior to corneal refractive surgery; *K*_post_ is keratometric value after corneal refractive surgery.

The authors do not provide data to support the reliability of their method.

#### 3.1.4. Knowledge of the Preoperative Keratometry and Refraction

Walter et al. [60] recommend using RPRE (patient refraction before surgery) as RX_TARG_ (i.e., the target refraction) utilizing AL (axial length) and *K*_PRE_ (i.e., keratometry before surgery).

The authors tested this method in 9 eyes undergoing IOL implantation after cataract surgery, obtaining a mean refractive error of +0.03*D* ± 0.42*D* ranging from −0.625*D* to +0.75*D*; 8 eyes where in the range of ±0,5*D* and 9 eyes in the range of ±1*D*.

#### 3.1.5. Knowledge of the Prerefractive Surgery Refraction

Latkany et al. [61] suggested different methods, among which is the use of the flattest keratometry value to calculate the IOL power, using the SRK-T formula:

(18) $$\begin{array}{c} \text{IOL}\;\text{implanted}=\text{IOL}\;\text{calc}-\left(0.47x+0.85\right), \end{array}$$

where *x* is preoperative refractive error.

However, since they found a hypocorrection with this method, they suggest modifying the result taking into account the preoperative refractive error.

### 3.2. Methods That Do Not Require the Knowledge of the Patient's Clinical History

Borasio et al. [62] suggested measuring the corneal thickness and the anterior and posterior corneal power with a Pentacam, together with a corneal refractive index, related to the corneal thickness. These data should be inserted in the so-called BESSt formula, to calculate the *K* values to be used with the SRK/T or with the Hoffer *Q* formula, depending on the axial length:

(19) BESSt  K ={[1rF∗(nadj−nair)]+[1rB∗(nacq−nadj)]    −[d∗1r∗(nadj−nair)∗1rB∗(nacq−nadj)]}  ∗1000,

where *rF* is anterior radius of curvature, *n*_adj_ is refractive index modified according to corneal thickness, *n*_air_ is refractive index of air (1), *n*_acq_ is aqueous index of refraction (1.336), and *rB* is posterior radius of curvature; *d* = *d*_cct_/1.3265; *d*_cct_ = CCT/1000000; CCT is central corneal thickness.

The authors tested their formula in 13 eyes (7 myopic, 6 hyperopic) undergoing IOL implantation after cataract, with a mean postoperative refractive error of 0.08*D* ± 0.62*D*, with 46% of the eyes in the range of ±0.5*D* and 100% in the range of ±1*D*.

Ferrara et al. [63], assuming that the index of refraction changes in relation with the corneal refractive treatment, proposed a second-order regression formula based on the axial length, to calculate a new index of refraction to be used to calculate the *K* values:

(20) $$\begin{array}{c} \text{IR}=-0.0006*\left(\mathrm{AL}*\mathrm{AL}\right)+0.0213*\mathrm{AL}+1.1572, \end{array}$$

where IR is the index of refraction and AL is the axial length.

The authors tested the formula in 5 eyes with the following results: ±0.50*D* in 2 eyes, ±1.00*D* in 4 eyes, and ±1.50*D* in 5 eyes (range −0.25*D* to −1.50*D*).

Haigis [64] suggested a formula to calculate the corrected corneal radius that is inversely related to the one measured with the IOL Master:

(21) $$\begin{array}{c} r_{\text{corr}}=\frac{331.5}{\left(-5.1625\times r_{\mathrm{meas}}+82.2603-0.35\right)}, \end{array}$$

where *r*_corr_ is corrected radius of curvature and *r*_meas⁡_ is radius of curvature after corneal refractive surgery measured with the IOL Master.

The author tested the formula in 117 eyes undergoing IOL implant after cataract surgery with a mean postoperative refractive error of −0.04*D* ± 0.7 ranging from −2.3*D* to +2.4*D*, with 61 eyes in the range of ± 0, 5*D*, 84 eyes in the range of ±1*D*, 98.4 eyes in the range of ±2*D*.

Ianchulev et al. [65] proposed a method which requires no special calculations; in fact, they suggested to perform phacoemulsification and measure the patient refraction on the operating table; this refraction, in terms of spherical equivalent, is multiplied for a constant, to obtain the IOL power to be implanted: IOL = 2.01449 × intraoperative spherical equivalent.

The authors tested the formula in 16 eyes undergoing IOL implantation after cataract extraction, and 83% of eyes were in the range of ±1*D*.

Kim et al. [66] proposed a formula in which the calculated corneal power is derived from a linear correlation with the mean corneal power after refractive surgery:

(22) $$\begin{array}{c} K_{M}=0.715*K_{C}+11.998, \end{array}$$

where *K*_*M*_ is average corneal power measured after refractive surgery. *K*_*C*_ is average corneal power recalculated by the formula.

The authors do not provide data to support the reliability of their method.

Latkany et al. [61] suggested different methods; among these they suggested to use the flattest keratometry value to calculate the IOL power, together with the SRK-T formula:

(23) $$\begin{array}{c} \text{IOL}\;\text{implanted}=\text{IOL}\;\text{calc}-\left(0.47x+0.85\right), \end{array}$$

where *x* is preoperative refractive error.

However, since they found a hypocorrection with this method, they suggest modifying the result utilizing also the preoperative refractive error.

The authors do not provide data to support the reliability of their method.

Mackool and Ko [67] suggested to perform phacoemulsification and measure the refraction 30 minutes later; the patient is then brought to the operating room for secondary IOL implantation. The IOL power is calculated multiplying the obtained refraction for a constant:

(24) $$\begin{array}{c} P=1.75*\text{AR}, \end{array}$$

where *P* is IOL power to be implanted and AR is aphakic refraction.

The authors tested the formula in 12 eyes undergoing IOL implantation after cataract surgery, with a mean postoperative refractive error of −0.3125 ± 1.15*D* ranging from −1.125*D* to +0.5*D*.

Rosa et al. [68, 69] were the first authors to publish a method that does not require the knowledge of the clinical history for the calculation of the IOL power in patients after excimer laser refractive surgery. The method is based on the following formula:

(25) $$\begin{array}{c} K_{\text{eff}}=\frac{337.5}{R_{\text{mis}}}*\left(0.0276*\mathrm{AL}+\;0.3635\right), \end{array}$$

where *K*_eff_ is corneal power to be included in calculation formulas, *R*_mis_ is patient mean radius of curvature measured with a common keratometry, and AL is axial length.

They suggest to use the *K*_eff_ with SRK T formula in eyes with axial length <30 mm and an average between the values of SRKT and SRK II in eyes with axial length over 30 mm.

The formula was tested in 62 eyes undergoing IOL implantation after cataract surgery with a mean postoperative refractive error of −0.41 ± 0.75*D* with a range running from −3.25*D* to +1*D*, with 37 eyes (60%) in the range of ±0.5*D*, 53 eyes (85%) in the range of ±1*D*, and 61 eyes (98%) in the range of ±2*D*.

The same authors, recently, proposed a modification to this method [70]: the power of the IOL should be calculated using the SRKT formula for all axial lengths utilizing the correction factor described above, but in case the product of AL (axial length) ∗  *K*_mis_ (corneal power measured with a common keratometer) is >1060, the IOL power obtained shall be reduced using the following regression formula:

(26) $$\begin{array}{c} Y=-\left(-0.0157*\mathrm{AL}*K_{\text{mis}}+16.437\right), \end{array}$$

where *Y* is refractive error to insert in the IOL power calculation to obtain emmetropia.

(For instance, if *Y* = 5, to obtain emmetropia, the calculation should aim to +5*D*.)

This new formula is more reliable than the first because it takes into account any hypocorrection or regressions that may be present in these patients after cornea refractive surgery.

Saiki et al. [71] proposed a modified double *K* method, also called anterior posterior (*A*-*P*) method, in patients that underwent laser in situ Keratomileusis (LASIK), utilizing a linear regression formula that requires the postoperative posterior corneal power evaluated with the Pentacam to calculate the preoperative Km to be used to calculate the ELP, similarly to the double *K* method:

(27) $$\begin{array}{c} y=-4.907x+12.371, \end{array}$$

where *y* is preoperative Km evaluated with the Pentacam and *x* is posterior postoperative Km evaluated with the Pentacam.

The authors tested their formula in 28 eyes of 19 patients: the median values of the arithmetic and absolute prediction errors using the *A*-*P* method were 0.16*D* and 0.54*D*, respectively. The prediction errors were within ±0.50*D* in 46.4% of eyes and within ±1.00*D* in 75.0%.

Savini et al. [72] suggested to calculate the corneal power utilizing a refractive index deriving from a regression formula related to the attempted correction:

(28) $$\begin{array}{c} P^{\text{post}}=\frac{\left(n^{\text{post}}-1\right)}{r}, \end{array}$$

where *P* ^post^ is the corneal power after corneal refractive surgery, *n* ^post^ is the postoperative index of refraction = 1.338 + 0.0009856 ∗ attempted correction, and *r* is radius of curvature.

The formula is the result of a retrospective analysis of 98 eyes that underwent myopic refractive surgery, utilizing the TMS 2 corneal topographer. The authors do not provide data to support the reliability of their method.

Shammas et al. [73, 74] proposed a formula in which the calculated mean corneal power is derived from a linear correlation with the mean corneal power after refractive surgery:

(29) $$\begin{array}{c} \;K_{\text{c}.\text{cd}}=1.14*K_{\text{post}}-6.8, \end{array}$$

where *K*_c.cd_ is mean corneal power recalculated by the formula and *K*_post_ is mean corneal power after refractive surgery.

The formula was tested in 15 eyes undergoing IOL implantation after cataract surgery, with a mean postoperative refractive error of 0.55 ± 0.31*D* ranging from −0.89*D* to +1.05*D* with 14 eyes (93.3%) in the range of ±1*D*.

Soper and Goffman [75] described the contact lens method, later modified by Holladay, which consists of three phases: measurement of refraction in diopters before contact lens application, application of a neutral contact lens with known curvature, and measurement of the refraction with the contact lens. After measuring the refraction before and after application of the contact lens, the change in refraction is added to the value of the contact lens curvature.

This method is slightly less accurate than the standard keratometry for determining corneal power in people with normal and transparent corneas, and with good visual acuity, and can be used in patients whose preoperative parameters are unknown, but unfortunately it is not reliable in case of media opacities which reduce the visual acuity below 20/70 and it is suggested after radial keratotomy but not after excimer laser corneal refractive surgery.

## 4. Conclusion

It is said that when there are too many ways to solve a problem it means that none of them is reliable. We think the real problem is that so far, as it is evident from the above, only few methods have been tested in a sufficient number of patients, while most of them are just theoretical and have been verified in few patients; in many of them, in fact, the studied group does not reach the number of twenty subjects.

The other problem is that several methods are clinical history based and, unfortunately, in most of the patients the preoperative keratometry values and the exact refractive treatment are not available, so we can conclude that methods that require knowledge of medical history are difficult to use.

With some of these methods, the authors report to have achieved 100% results in the range of 1 diopter, but we believe this is only due to the limited number of patients tested: in fact, even in patients without a story of previous refractive surgery, these results would be described as impressive. If these methods were in fact so precise, it would be probably convenient to treat them first with refractive surgery and later to perform the cataract surgery, but obviously this is illogical. Therefore we recommend, to avoid postoperative nasty surprises, using only those methods that have shown good results in a large number of patients, possibly by averaging the results obtained with these methods.
